# Milk-y Way: the impact of single-nucleotide polymorphisms on milk production traits in Kilis dairy goats

**DOI:** 10.5194/aab-66-369-2023

**Published:** 2023-11-24

**Authors:** Zühal Gündüz, Osman Biçer

**Affiliations:** 1 Department of Agricultural Biotechnology, Faculty of Agriculture, Adnan Menderes University, Aydın, Türkiye; 2 Department of Animal Science, Faculty of Agriculture, Hatay Mustafa Kemal University, Hatay, Türkiye

## Abstract

This study aimed to investigate the impact of single-nucleotide polymorphisms (SNPs) on milk production traits in Kilis dairy goats by analyzing the genotypes of *POU1F1*, *PRLR*, β *-Lg*, *GH1*, and *GH2* genes and their association with lactation milk yield (LMY), lactation length (LL) and average daily milk yield (ADMY). Blood samples were collected from 227 goats, and genotyping was performed using polymerase chain reaction restriction fragment length polymorphism (PCR-RFLP). The results revealed that the frequencies of the genotypes varied among the genes. The polymorphisms were found to be significantly linked with milk production traits. These findings suggest that SNPs of *POU1F1/AluI*, *PRLR/RsaI* and β *-Lg/SacII* are significantly associated with LMY and that the *POU1F1-TC* genotype, *PRLR-TT* genotype and β *-Lg-AB* genotype are associated with higher LMY and ADMY. Additionally, the *POU1F1-TC* genotype was found to have a longer LL. However, no significant association was found between the *GH1* and *GH2* genotypes and LMY, LL and ADMY. Overall, this study provides valuable insights into the genetic factors influencing milk production traits in Kilis dairy goats, which can be utilized for the selection of high-yielding animals in breeding programs.

## 1 Introduction

The accurate selection of dairy goats with superior milk production traits is essential for breeders and consumers. While traditional selection methods have been used in the past, new approaches, such as the use of genetic markers, have the potential to enhance the precision and efficiency of selection programs. Genetic polymorphisms, in particular, have been closely linked to desired traits of interest, making them a valuable tool for breeders and researchers. Identification and utilization of these genetic markers can improve the overall performance of dairy goats and facilitate the selection of animals with desirable traits for breeding programs. Therefore, continued research on genetic polymorphisms in dairy goats is necessary to improve the selection process and meet the increasing demand for high-quality dairy products.

Goat husbandry in Türkiye plays a pivotal role in the field of animal production (Gül et al., 2020; Gursoy, 2006), with an approximate population of 12 325 000 goats (TUIK, 2022). However, traditional breeding programs are limited with respect to achieving genetic progress, which can take several generations. The use of major genes can accelerate genetic progress, but this requires an understanding of the effect levels of these genes, knowing individual genotypes and developing appropriate mating plans. The Kilis goat has emerged as a breed with a unique genetic profile that reflects its complex ancestry due to historical crossbreeding between the Damascus and Hair goat populations in the region (Gül et al., 2020). The Kilis goat is well adapted to the arid and semiarid regions of Türkiye and is known for its high milk yield and fecundity. Therefore, it holds great potential for milk production and genetic improvement programs in Türkiye.

The *POU1F1* (POU class 1 homeobox 1) gene plays a critical role in the growth and development of mammals and is secreted from the anterior pituitary gland. *POU1F1* is involved in the regulation of various important physiological processes, including somatic growth, mammary gland development and lactation (Lan et al., 2009; Bastos et al., 2006). Genetic variations in the *POU1F1* gene have been found to be associated with variations in milk production traits in dairy goats, making it a promising target for genetic selection programs aimed at improving milk yield and quality (Işık, 2016; Daga et al., 2013; Mura et al., 2012). Prolactin (*PRL*) is a peptide hormone that plays a crucial role in the development and function of the mammary gland, including milk production and the expression of milk protein genes (Viitala et al., 2006). The biological effects of prolactin are exerted through its interaction with the prolactin receptor (*PRLR*). The genetic relationship between the *PRL* and *PRLR* loci provides valuable insights into the metabolic and functional aspects of the prolactin endocrine axis (Hou et al., 2013). In particular, variations in the *PRLR* gene have been linked to mammary gland development and differentiation as well as to milk production traits and milk composition in livestock species (Skrzypczak et al., 2015; Hou et al., 2014, 2013; Lü et al., 2011). β -lactoglobulin is a key whey protein found in ruminant milk and belongs to the lipocalin superfamily of hydrophobic molecule carriers. The gene encoding β -lactoglobulin is highly expressed during lactation, particularly in the mammary gland (Mercier and Vilotte, 1993). The expression of this gene is tightly regulated by a complex hormonal mechanism, with prolactin being the main lactogenic stimulus. The β *-Lg* gene has been found to have polymorphisms that are particularly associated with various milk production traits, such as milk quality, yield and composition, in different ruminant species (Yousefi et al., 2013; Erdoğan, 2010). The growth hormone (*GH*) gene is part of a large gene family that includes prolactin (*PRL*) and placental lactogens (CS, chorionic somatomammotropins) (Harvey et al., 1995). *GH* is specifically expressed from somatotrophic cells in the anterior pituitary gland, and its transcription is regulated by cell-type-specific hormonal mechanisms. Therefore, *GH* serves as a notable model for understanding the cell type-specific control mechanisms of gene expression during organ development. As the name implies, *GH* is a crucial hormone for growth and development, and it is also involved in the processes of sexual differentiation and pubertal maturation. *GH* also participates in gonadal steroidogenesis, gametogenesis and ovulation, and it influences pregnancy and lactation (Hull and Harvey, 2001). Thus, a better understanding of the functional significance of the these genes in relation to milk production traits can provide valuable insights for the development of effective breeding strategies in dairy goat farming.

This study aimed to investigate polymorphisms in the *POU1F1*, *PRLR*, β *-Lg* and *GH* genes in Kilis goats as well as their relationships with lactation milk yield, lactation length and average lactation milk yield. These polymorphisms can serve as useful genetic markers for animal breeding via marker-assisted selection (MAS).

## 2 Material and methods

### 2.1 Animal selection, data collection and DNA sampling procedure

The Kilis and Damascus (Shami) goat breeds are renowned for their notable milk production and reproductive attributes. These breeds are primarily reared in the provinces of Kilis, Gaziantep, Hatay and Şanlıurfa, situated in the southern region of Türkiye.

It is thought that Kilis goats originated from the uncontrolled mating of Damascus (Shami) goats from Syria and Hair goats, which are one of the domestic goat breeds in Türkiye, under conventional breeding conditions (Gül et al., 2020; Keskin et al., 2017). The unrelated animal subjects of the study comprised 227 Kilis goats, which were obtained from the National Breeding Project of Kilis Goats under Farm Conditions. The project was supported by the General Directory of Agricultural Research and Policy of the Republic of Türkiye’s Ministry of Agriculture and Forestry in Kilis Province. All goats were aged between 2 and 4 years and raised under uniform management and feeding conditions. Individual and total milk yields were recorded every 28 d until the end of lactation. Blood samples were collected from the vena jugularis of each dairy goat using vacuum tubes containing anticoagulant ethylenediaminetetraacetic acid (BD Vacutainer Systems, Plymouth, UK). Approximately 9 mL of blood was collected from each animal. The genomic DNA was extracted from white blood cells using the high-salt method procedure (Miller et al., 1988) and was stored at -20 ${}^{\circ }$ C until use.

### 2.2 Polymerase chain reaction (PCR) reactions and genotyping

In this study, two pairs of primers were designed to amplify the goat *POU1F1*, *PRLR*, β *-Lg*, *GH1* and *GH2* genes. Table 1 provides detailed information on the primer sequences and their corresponding products.

**Table 1:** The primer sequences and information on the genes.

Primer	Primer sequence	GenBank ID	Region	Product size
*POU1F1_F*	5 ${}^{\prime }$ -CCATCATCTCCCTTCTT-3 ${}^{\prime }$	MH892432.1	Intron 5, exon 6, 3 ${}^{\prime }$ flanking	450 bp
*POU1F1_R*	5 ${}^{\prime }$ -AATGTACAATGTGCCTTCT-3 ${}^{\prime }$			
*PRLR_F*	5 ${}^{\prime }$ -AGTGAGAGTTATGGAAGGATG-3 ${}^{\prime }$	KJ572972.1	3 ${}^{\prime }$ UTR	443 bp
*PRLR_R*	5 ${}^{\prime }$ -AAGGTTAAGCAACTGGTCTT-3 ${}^{\prime }$			
β *-Lg_F*	5 ${}^{\prime }$ -CGGGAGCCTTGGCCCCTCTG-3 ${}^{\prime }$	Z33881.1	Exon 7, 3 ${}^{\prime }$ UTR	427 bp
β *-Lg_R*	5 ${}^{\prime }$ -CCTTTGTCGAGTTTGGGTGT-3 ${}^{\prime }$			
*GH1_F*	5 ${}^{\prime }$ -CTCTGCCTGCCCTGGACT-3 ${}^{\prime }$	D00476.1	Exon 2, intron 2, exon 3	422 bp
*GH1_R*	5 ${}^{\prime }$ -GGAGAAGCAGAAGGCAACC-3 ${}^{\prime }$			
*GH2_F*	5 ${}^{\prime }$ -TCAGCAGAGTCTTCACCA AC-3 ${}^{\prime }$	D00476.1	Exon 4	116 bp
*GH2_R*	5 ${}^{\prime }$ -CAACAACGCCATCCTCAC-3 ${}^{\prime }$			

UTR denotes untranslated regions and “bp” stands for base pairs.

The PCR reactions were carried out using genomic DNA and specific concentrations of reagents (Table 2). The thermal cycling protocols involved denaturation, annealing and extension steps, which varied with respect to temperature and time for each gene. The PCR amplification was performed using the C1000 Touch™ thermal cycler (Bio-Rad, USA). To analyze the PCR products of the *POU1F1*, *PRLR*, β *-Lg* and *GH* genes, aliquots of 30 µ L were digested with 10 units of restriction enzymes AluI, RsaI, SacII and HaeIII (Thermo Scientific, USA) for 2 h at 37 ${}^{\circ }$ C, according to the supplier's instructions. The resulting digested products were then visualized by electrophoresis in a 2.0 % agarose gel stained with ethidium bromide.

**Table 2:** PCR reactions and the cycling protocol.

Reaction	*POU1F1*	*PRLR*	β *-Lg*	*GH1*	*GH2*
10× buffer	1×	1×	1×	1×	1×
MgCl2	2 mM	2 mM	1.5 mM	2 mM	1.5 mM
dNTP (ABM)	200 µ M	200 µ M	200 µ M	200 µ M	200 µ M
Forward primer	0.5 µ M	1 µ M	0.5 µ M	0.6 µ M	0.6 µ M
Reverse primer	0.5 µ M	1 µ M	0.5 µ M	0.6 µ M	0.6 µ M
Taq DNA	0.625 U ${}^{a}$	1 U ${}^{b}$	0.625 U ${}^{a}$	1 U ${}^{b}$	1 U ${}^{b}$
Genomic DNA	∼ 100 ng	∼ 100 ng	∼ 100 ng	∼ 100 ng	∼ 100 ng
Total volume	30 µ L	30 µ L	30 µ L	30 µ L	30 µ L
PCR cycling	95 ${}^{\circ }$ C for 5 min,	95 ${}^{\circ }$ C for 5 min,	95 ${}^{\circ }$ C for 5 min,	95 ${}^{\circ }$ C for 5 min,	95 ${}^{\circ }$ C for 5 min,
protocol	36 cycles at	35 cycles at	35 cycles at	36 cycles at	42 cycles at
	94 ${}^{\circ }$ C for 45 s, 52 ${}^{\circ }$ C	94 ${}^{\circ }$ C for 40 s, 51.4 ${}^{\circ }$ C	95 ${}^{\circ }$ C for 40s, 60.9 ${}^{\circ }$ C	95 ${}^{\circ }$ C for 30 s, 63 ${}^{\circ }$ C	94 ${}^{\circ }$ C for 30 s, 54 ${}^{\circ }$ C
	for 45 s, 68 ${}^{\circ }$ C for 1 min	for 40 s, 72 ${}^{\circ }$ C for 1 min	for 1 min, 68 ${}^{\circ }$ C for 90 s	for 30 s, 72 ${}^{\circ }$ C for 45 s	for 30 s, 72 ${}^{\circ }$ C for 45 s

${}^{a}$ The Taq DNA polymerase enzyme and buffer from New England Biolabs (NEB), with the amount utilized expressed as units (U), were used. ${}^{b}$ The Taq DNA polymerase enzyme and buffer from Thermo Scientific USA, with the amount utilized expressed as units (U), were used.

### 2.3 Statistical analysis

In this study, the goats utilized were granted unrestricted access to water. The lactation milk yield was determined through regular milk control measures taken every 28 d. The AT method from the International Committee for Animal Recording (ICAR) was employed to estimate the lactation milk yield, utilizing the following equation:

MY=IMY⋅(TMF/TF),

where MY denotes the milk yield of goats on the control day, IMY refers to the individual milk yield of each goat during morning milking, TMF indicates the total milk yield of all animals during morning and evening milking, and TF represents the total milk yield of all animals during morning milking. In previous studies, Gül et al. (2016) employed the Fleishman method to calculate the milk yield of each goat.

To establish a relationship between animal genotypes and lactation milk yield, the general linear model of SPSS (Kinnear and Gray, 1999) was used, with lactation length and average daily lactation milk yield serving as parameters. The model was as follows:

$$Y_{ij}=\mu +\alpha_{i}+e_{ij},$$

where $Y_{ij}$ represents the trait measured on the ij th animal, μ refers to population means, $\alpha_{i}$ denotes the effect of the i th genotype and $e_{ij}$ represents the effect of random error.

The experimental cohort comprised animals that had given birth either twice or three times. When calculating milk yields, values were standardized based on the overall average. Additive correction factors were applied to reduce variations caused by differences in the number of births, birth type, and farms

Allelic frequencies, genotyping frequencies and Hardy–Weinberg equilibriums were calculated using the GenAlEx 6.5 (Peakall and Smouse, 2012) and POPGENE 32 (Yeh et al., 2000) software packages.

## 3 Results

### 3.1 Genotyping

Electrophoretic analysis of DNA patterns resulting from AluI endonuclease restriction of the goat *POU1F1* gene (using the GeneRuler Low Range DNA Ladder from Thermo Scientific) revealed three distinct genotypes: *CC*, *TT*, and *TC*. The *CC* genotype exhibited fragments of sizes 216, 124 and 110 bp. The *TT* genotype displayed fragments of sizes 340 and 110 bp, while the *TC* genotype manifested fragments of sizes 340, 216, 124 and 110 bp (Lan et al., 2007). Due to challenges associated with the precise resolution of the 110 bp fragment on a 2 % agarose gel, it was observed that genotypes *CC* and *TC* presented with two to three fragments that were imperceptible under visual inspection. Notably, the fragments spanning 340 and 216 bp demonstrated robust visibility and efficacy in accurately delineating the genotypic variations.

After digestion with RsaI endonuclease of the goat *PRLR* gene (GeneRuler 50 bp DNA Ladder, Thermo Scientific), the *CC* (383 and 60), *CT* (443, 383 and 60) and *TT* (443) genotypes were obtained as a result of the reaction of PCR products with the restriction endonuclease enzyme (Hou et al., 2014). Due to challenges associated with the precise resolution of the 60 bp fragment on a 2 % agarose gel, it was observed that genotypes *CC* and *CT* presented with one or two fragments that were imperceptible under visual inspection. Notably, the fragments spanning 443 and 383 bp demonstrated robust visibility and efficacy in accurately delineating the genotypic variations.

After digestion with SacII endonuclease of the goat β *-Lg* gene (GeneRuler 50 bp DNA Ladder, Thermo Scientific), the *AA* (427), *AB* (427, 349 and 78) and *BB* (349 and 78) genotypes were obtained as a result of the reaction of PCR products with restriction endonuclease enzyme (Pena et al., 2000). Due to challenges associated with the precise resolution of the 78 bp fragment on a 2 % agarose gel, it was observed that genotypes *AB* and *BB* presented with one or two fragments that were imperceptible under visual inspection. Notably, the fragments spanning 427 and 349 bp demonstrated robust visibility and efficacy in accurately delineating the genotypic variations.

After digestion with HaeIII endonuclease of the goat *GH1* gene (GeneRuler 50 bp DNA Ladder, Thermo Scientific), the *AA* (366 and 56), *AB* (422, 366 and 56) and *BB* (422) genotypes were obtained as a result of the reaction of PCR products with restriction endonuclease enzyme (Hua et al., 2009). Due to challenges associated with the precise resolution of the 76 bp fragment on a 2 % agarose gel, it was observed that genotypes *AA* and *AB* presented with two or three fragments that were imperceptible under visual inspection. Notably, the fragments spanning 422 and 366 bp demonstrated robust visibility and efficacy in accurately delineating the genotypic variations. The *BB* genotype was not found in Kilis goats in this study.

After digestion with HaeIII endonuclease of the goat *GH2* gene (GeneRuler Low Range DNA Ladder, Thermo Scientific), the *CC* (88 and 28), *CD* (116, 88 and 28) and *DD* (116) genotypes were obtained as a result of the reaction of PCR products with restriction endonuclease cenzyme (Hua et al., 2009). Due to challenges associated with the precise resolution of the 28 bp fragment on a 2 % agarose gel, it was observed that the genotype *CD* presented with one fragment that was imperceptible under visual inspection. Notably, the fragments spanning 116 and 88 bp demonstrated robust visibility and efficacy in accurately delineating the genotypic variations. The *CC* genotype was not found in Kilis goats in this study.

**Figure 1:**
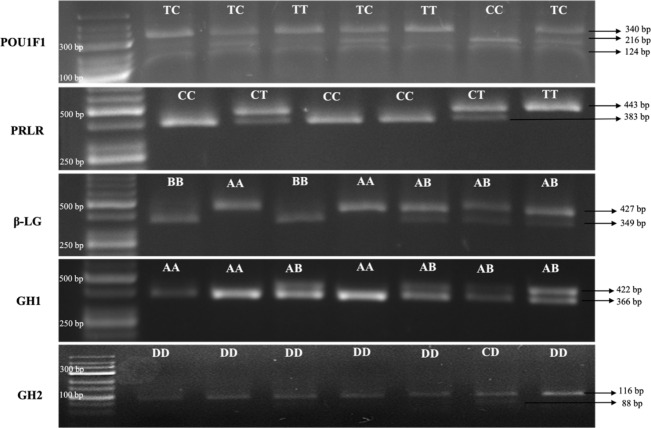
Electrophoretic profile for the *POU1F1*, *PRLR*, β *-Lg*, *GH1* and *GH2* fragments.

Figure 1 illustrates the fragments of DNA that have undergone cleavage by restriction enzymes in the study.

### 3.2 Allelic and genotypic frequencies of the genes

The present study utilized the polymerase chain reaction restriction fragment length polymorphism (PCR-RFLP) method to detect allelic and genotypic frequencies of various genes, including *POU1F1/AluI*, *PRLR/RsaI*, β *-Lg/SacII*, *GH1/HaeIII* and *GH2/HaeIII*, in caprine genomic DNA. The results are presented in Table 3, where the frequencies of different alleles for each locus were determined.

**Table 3:** Allelic and genotyping frequencies of the genes.

Locus	Allele frequencies		Genotype frequencies			$H_{o}$	$H_{e}$	$\chi^{2}$
	*T*	*C*	*TT*	*TC*	*CC*			
*POU1F1*	0.671	0.329	0.408	0.525	0.067	0.525	0.442	4.276 ${}^{*}$
	*C*	*T*	*CC*	*CT*	*TT*			
*PRLR*	0.868	0.132	0.754	0.224	0.022	0.219	0.229	0.345 ${}^{\mathrm{ns}}$
	*A*	*B*	*AA*	*AB*	*BB*			
β *-Lg*	0.590	0.410	0.303	0.579	0.118	0.582	0.484	7.306 ${}^{**}$
	*A*	*B*	*AA*	*AB*	*BB*			
*GH1*	0.551	0.449	0.102	0.898	–	0.898	0.495	117.679 ${}^{***}$
	*C*	*D*	*CC*	*CD*	*DD*			
*GH2*	0.008	0.992	–	0.017	0.983	0.017	0.016	0.013 ${}^{\mathrm{ns}}$

Observed ( $H_{o}$ ) and expected ( $H_{e}$ ) heterozygosity and the chi-square ( $\chi^{2}$ ) statistic. The following levels of significance are utilized: ${}^{*}$ P<0.05 , ${}^{**}$ P<0.01 , ${}^{***}$ P<0.001 and non-significant (ns).

For the *POU1F1/AluI* locus, the *T* and *C* allele frequencies were 0.671 and 0.329, respectively. The *C* and *T* allele frequencies for the *PRLR/RsaI* locus were 0.868 and 0.132, respectively. Similarly, the *A* and *B* allele frequencies for the β *-Lg/SacII* locus were 0.590 and 0.410, respectively. For the *GH1/HaeIII* locus, the *A* and *B* allele frequencies were 0.551 and 0.449, respectively, and no *BB* genotype was observed. For the *GH2/HaeIII* locus, the *C* and *D* allele frequencies were 0.008 and 0.992, respectively, and no *CC* genotype was observed. Furthermore, all genotype distributions for the loci mentioned above were found to be at Hardy–Weinberg equilibrium, except for *POU1F1/AluI* ( P<0.05 ), β *-Lg/SacII* ( P<0.01 ) and *GH1/HaeIII* ( P<0.001 ).

The method from Nei et al. (1983) was used to determine the observed heterozygosity ( $H_{o}$ ) and expected heterozygosity ( $H_{e}$ ) values for each locus. For the *POU1F1-AluI* locus, the $H_{o}$ and $H_{e}$ values were 0.525 and 0.442, respectively. Similarly, for the *PRLR/RsaI* locus, the $H_{o}$ and $H_{e}$ values were 0.219 and 0.229, respectively. The $H_{o}$ and $H_{e}$ values for the β *-Lg/SacII* locus were 0.582 and 0.484, respectively. For the *GH1/HaeIII* locus, the $H_{o}$ and $H_{e}$ values were 0.898 and 0.495, respectively. Lastly, for the *GH2/HaeIII* locus, the $H_{o}$ and $H_{e}$ values were 0.017 and 0.016, respectively.

### 3.3 Genetic variations and milk performance in goat populations

Table 4 presents the effects of the *POU1F1*, *PRLR*, β *-Lg*, *GH1* and *GH2* genotypes on lactation traits in Kilis goats. The results showed that individuals with the *TC* genotype in the *POU1F1* locus had a considerably ( P=0.012 ) higher lactation milk yield than those with the *TT* genotype (579.55 ± 183.62 kg vs. 470.45 ± 175.86 kg, respectively), but there was no difference between *TT* vs. *CC* or *TC* vs. *CC*. Similarly, animals with the *TC* genotype had a significantly ( P=0.021 ) longer lactation length than animals with the *TT* genotype (254.35 ± 10.78 d vs. 243.88 ± 14.69 d, respectively), but there was no difference between *TT* vs. *CC* or *TC* vs. *CC*. Furthermore, Kilis goats with the *TC* genotype had a significantly ( P=0.022 ) higher average daily milk yield than animals with the *TT* genotype (2.27 ± 0.68 kg vs. 1.90 ± 0.66 kg, respectively), but there was no difference between *TT* vs. *CC* or *TC* vs. *CC*.

Regarding the *PRLR* locus, individuals with the *TT* genotype had a significantly ( P=0.050 ) greater lactation milk yield than those with the *CC* and *CT* genotypes (691.22 ± 220.66 vs. 453.98 ± 186 and 449.22 ± 207.20 kg, respectively). The same pattern was observed for the average daily milk yield (2.71 ± 0.86 vs. 1.82 ± 0.71 and 1.78 ± 0.76 kg, respectively). There was no significant difference between the *TT*, *TC* and *CC* genotypes for lactation length; however, Kilis goats with the *TT* genotype generally had longer lactation length than animals with the *TC* and *CC* genotypes.

With respect to the β *-Lg* locus, individuals with the *AB* genotype had a significantly ( P=0.001 ) higher lactation milk yield than those with the *BB* genotypes (485.65 ± 209.64 vs. 308.26 ± 141.20 kg, respectively), but there was no difference between the *AA* and *AB* genotypes. The same pattern was observed for the average daily milk yield (1.93 ± 0.79 vs. 1.30 ± 0.54, respectively). There was no significant difference between the *AA*, *AB* and *BB* genotypes for lactation length; however, animals with the *AB* genotype generally had longer lactation length than those with *AA* and *BB* genotypes.

The *AB* genotype in the *GH1* locus showed a higher lactation milk yield, lactation length and average daily milk yield than the *AA* genotype, but the differences were not statistically significant. Moreover, the *CD* genotype in the *GH2* locus exhibited a higher lactation milk yield, lactation length and average daily milk yield than the *DD* genotype, but the differences were statistically insignificant.

**Table 4:** The effect of the genotype (least square means ± standard deviation) on the lactation milk yield (LMY, in kg), lactation length (LL, in days) and average daily milk yield (ADMY in kg).

Genotype		LMY	LL	ADMY
*POU1F1*	N	P = 0.012	P = 0.021	P = 0.022
*TT*	49	470.45 ± 175.86 ${}^{a}$	243.88 ± 14.69 ${}^{a}$	1.90 ± 0.66 ${}^{a}$
*TC*	63	579.55 ± 183.62 ${}^{b}$	254.35 ± 10.78 ${}^{b}$	2.27 ± 0.68 ${}^{b}$
*CC*	8	489.62 ± 145.45 ${}^{\mathrm{ab}}$	249.75 ± 12.59 ${}^{\mathrm{ab}}$	1.95 ± 0.56 ${}^{\mathrm{ab}}$
Mean	120	535.56 ± 173.49	251.76 ± 12.11	2.11 ± 0.65
*PRLR*	N	P = 0.050	P = 0.882	P = 0.048
*CC*	135	453.98 ± 186.07 ${}^{a}$	250.79 ± 64.25	1.82 ± 0.71 ${}^{a}$
*CT*	40	449.22 ± 207.20 ${}^{a}$	245.95 ± 17.02	1.78 ± 0.76 ${}^{a}$
*TT*	4	691.22 ± 220.66 ${}^{b}$	254.25 ± 13.74	2.71 ± 0.86 ${}^{b}$
Mean	179	458.22 ± 193.77	249.78 ± 56.38	1.83 ± 0.73
β *-Lg*	N	P = 0.001	P = 0.284	P = 0.001
*AA*	54	475.00 ± 159.60 ${}^{a}$	248.09 ± 13.25	1.91 ± 0.62 ${}^{a}$
*AB*	103	485.65 ± 209.64 ${}^{a}$	254.04 ± 72.97	1.93 ± 0.79 ${}^{a}$
*BB*	21	308.26 ± 141.20 ${}^{b}$	232.81 ± 16.14	1.30 ± 0.54 ${}^{b}$
Mean	178	461.49 ± 195.93	249.73 ± 56.53	1.84 ± 0.74
*GH1*	N	P = 0.168	P = 0.529	P = 0.213
*AA*	18	399.87 ± 138.84	241.94 ± 15.03	1.63 ± 0.51
*AB*	159	467.43 ± 201.43	250.85 ± 59.58	1.86 ± 0.76
Mean	177	460.56 ± 196.74	249.94 ± 56.71	1.83 ± 0.74
*GH2*	N	P = 0.557	P = 0.955	P = 0.546
*CD*	3	525.80 ± 97.96	251.67 ± 12.01	2.09 ± 0.43
*DD*	178	459.05 ± 195.79	249.82 ± 56.54	1.83 ± 0.74
Mean	181	460.15 ± 194.62	249.85 ± 56.08	1.84 ± 0.73

N : number of animals. Different letters in the same column indicate significant differences.

## 4 Discussion

In the current study, the *TC* genotype of *POU1F1* demonstrated superior values across all examined traits, whereas the *TT* genotype exhibited the lowest values. The differences observed among genotypes were statistically significant for the LMY ( P = 0.012), LL ( P = 0.021) and ADMY ( P = 0.022) in Kilis goats. Our findings align with previous research, which has also reported a greater LMY in goats with the *TC* genotype (Işık, 2016; Lan et al., 2009, 2007). Previous studies have also reported a relationship between different parts of the *POU1F1* gene and milk yield in various animal species, including goats, sheep and cattle. Zhou et al. (2016) found an association between the *POU1F1/AluI* locus and the LMY, while other authors have reported associations between different parts of the *POU1F1* gene and the LMY (Özmen et al., 2014; Lan et al., 2013; Mura et al., 2012) and milk production traits in cattle (Khaizaran and Al-Razem, 2014). However, in the study conducted by Huang et al. (2008), a significant effect of the *POU1F1* gene on the LMY was observed only for exon 3, whereas no effect was observed for intron 5 nor exon 6 (Trakovicka et al., 2014; Yan et al., 2011). In our study, the *TC* genotype of *POU1F1* was associated with a longer lactation length compared with the *CC* and *TT* genotypes. Although studies on lactation period are limited, some other studies have also reported significant effects of the *POU1F1* gene on lactation length in sheep (Al-Khuzai and Al-Anbari, 2018) and cattle (Edriss et al., 2009). Moreover, we found a significant relationship between genotypes and the ADMY, with the *TC* genotype showing a higher ADMY than the other genotypes.

The *TT* genotype of *PRLR* displayed the highest value for all evaluated traits. Significant differences were observed among the genotypes with respect to the LMY ( P = 0.050) and ADMY ( P = 0.048) but not the LL in Kilis goats. Although the differences between the *CC* and *CT* genotypes were not statistically significant for LL, our results indicate that the *TT* genotype has a positive effect on the LL. Recently, most studies have focused on the association of the *PRLR* gene with reproductive traits in different species, particularly in pigs. In their investigation, El-Shorbagy et al. (2022) underscored that individuals exhibiting the *CT* genotype displayed a notably elevated daily average milk yield as well as overall milk yield in comparison with those possessing the *TT* genotype in Egyptian Zaraibi goats. Hou et al. (2014) reported that the *PRLR/RsaI* locus had an impact on the LMY, but the *TT* genotype was not observed in the studied populations. In addition, they found that the *CC* genotype was associated with a higher lactation milk yield. However, numerous studies have reported associations between different genotypes from various regions of the *PRLR* gene and milk production traits in different species, including the promoter region of *PRLR* (Fontanesi et al., 2014), intron 2 and exon 9 (Hou et al., 2013), and exon 10 (Lü et al., 2011) in goats as well as exon 3 and exon 7 (Zhang et al., 2008) in cattle. To our knowledge, no studies have investigated the impact of *PRLR* genotypes on the LL and ADMY in Kilis goats or other species. Therefore, further studies are required to confirm our findings and to explore the role of *PRLR* genotypes in lactation performance across different breeds and species.

Our findings in Table 4 also reveal an association between genotypes and the tested milk production traits. Specifically, we observed that individuals with the *AB* genotype of β *-Lg* had the highest values of LMY, LL and ADMY. While the differences between the *AA* and *AB* genotypes were not statistically significant for the LL, we found a significant association between the *AB* genotype and the LMY ( P = 0.001) as well as the ADMY ( P = 0.001). Although the differences in the LL were not statistically significant, we did observe a positive effect of the *AA* and *AB* genotypes on the LL. This is in agreement with previous studies that also reported an effect of the β *-Lg* gene on the LMY in goats (Dettori et al., 2015). For instance, Kahilo et al. (2014) reported that individuals with the *AB* genotype had a greater LMY in Nubian goats, which is consistent with our findings. Similarly, other studies have reported that the *AA* genotype had a higher LMY than other genotypes in certain goat populations (El-Hanafy et al., 2015, 2010; Kahilo et al., 2014). However, there have also been studies showing no significant effect of the β *-Lg* gene on the LMY in Saanen and Pakistani domestic goat breeds (Işık et al., 2017; Mahmood et al., 2016). Similarly, in their study, Wardani et al. (2022) noted that there was no statistically significant difference in the association between milk yield and β -lactoglobulin in Senduro goats. Conversely, findings by Dokic et al. (2020) demonstrated a correlation between β -lactoglobulin ( β -Lg) polymorphism and milk performance in sheep. The study revealed the prevalence of the *BB* genotype over the other variants (*AA* and *AB*) in terms of milk yield, while no statistically significant disparities among these genotypes were observed across different sheep breeds. The research conducted by Wafaa et al. (2019) unveiled noteworthy distinctions in overall milk production; specifically, the genetic structures denoted as *AA* exhibited superiority over the genetic structures marked as *AB*, with a difference in the kilograms of total milk yield. However, no significant variances were observed between the two genetic structures concerning lactation duration. In Çine Çapari sheep, it has been reported that individuals with the *AA* genotype have a longer LL compared with other genotypes, but no similar studies have been conducted on goats in terms of the relevant locus (Erdoğan, 2010). Additionally, studies on the β *-Lg* gene in other regions have reported significant effects of the gene on the ADMY in buffalo (Vohra et al., 2012) but no significant effect on the ADMY in sheep (Triantaphyllopoulos et al., 2017).

Our statistical analysis revealed that there was no significant association between *GH1/HaeIII*, *GH2/HaeIII* and milk production traits. This finding is consistent with previous reports that demonstrated no relationship between *GH1* and milk yield in Algarvia goats (Malveiro et al., 2001). Likewise, *GH/TaqI* was also found to be insignificant, and the *GH/AluI* locus was monomorphic in Kankrej cattle (Falaki et al., 1997). However, individuals with the *AB* genotype had a higher lactation milk yield in Serrana and Sarda goats (Dettori et al., 2013; Marques et al., 2003) as well as in cattle (Krasnopiorova et al., 2012).

Interestingly, *GH2* had a significant effect on the LMY in Algarvia and Serrana goats (Marques et al., 2003; Malveiro et al., 2001), but its effect was found to be insignificant in Sarda goats (Dettori et al., 2013). In our study, although the differences between genotypes were statistically insignificant, individuals with *AB* and *CD* genotypes had higher values, suggesting a positive effect on the LMY, LL and ADMY.

## 5 Conclusion

Our study suggests that the genetic variations present in the *POU1F1/AluI*, *PRLR/RsaI* and β *-Lg/SacII* loci are associated with milk production traits in dairy goats. This finding suggests that this markers may be useful for improving milk production traits in dairy goat breeding programs. Therefore, our results could provide a valuable reference for implementing MAS in the breeding and genetics of dairy goats, with the aim of improving milk production traits.

The results of our study provide important information for genetic improvement programs in the dairy goat industry. The identification of genetic markers associated with milk production traits can help breeders to select the best animals for breeding purposes, leading to more efficient and profitable dairy production. The use of MAS can facilitate the selection of superior individuals for milk production traits and can accelerate genetic progress in dairy goats. Therefore, our findings have important implications for the development of breeding strategies that focus on improving milk production traits in dairy goats.

However, it is worth noting that our study has some limitations, such as the small sample size and the focus on a single goat breed. Further studies with larger sample sizes and involving multiple goat breeds are needed to validate our findings and to investigate the generalizability of these genetic markers to other goat populations. Nevertheless, our study contributes to the understanding of the genetic basis of milk production traits in dairy goats and provides a basis for future studies in this area.

## Data Availability

In accordance with the principles of scientific transparency and reproducibility, the data used and analyzed in this study are available from the corresponding author upon reasonable request.
